# Dimensions of social categorization: Inside the role of language

**DOI:** 10.1371/journal.pone.0254513

**Published:** 2021-07-12

**Authors:** Anna Lorenzoni, Mikel Santesteban, Francesca Peressotti, Cristina Baus, Eduardo Navarrete

**Affiliations:** 1 Dipartimento di Psicologia dello Sviluppo e della Socializzazione, Università degli Studi di Padova, Padova, Italia; 2 Department of Linguistics and Basque Studies, University of the Basque Country (UPV/EHU), Vitoria-Gasteiz, Spain; 3 Department of Cognition, Development and Educational Psychology, University of Barcelona, Barcelona, Spain; CNRS - Université d’Aix-Marseille, FRANCE

## Abstract

The present pre-registration aims to investigate the role of language as a dimension of social categorization. Our critical aim is to investigate whether language can be used as a dimension of social categorization even when the languages coexist within the same sociolinguistic group, as is the case in bilingual communities where two languages are used in daily social interactions. We will use the memory confusion paradigm (also known as the Who said what? task). In the first part of the task, i.e. encoding, participants will be presented with a face (i.e. speaker) and will listen to an auditory sentence. Two languages will be used, with half of the faces always associated with one language and the other half with the other language. In the second phase, i.e. recognition, all the faces will be presented on the screen and participants will decide which face uttered which sentence in the encoding phase. Based on previous literature, we expect that participants will be more likely to confuse faces from within the same language category than from the other language category. Participants will be bilingual individuals of two bilingual communities, the Basque Country (Spain) and Veneto (Italy). The two languages of these communities will be used, Spanish and Basque (Study 1), and Italian and Venetian dialect (Study 2). Furthermore, we will explore whether the amount of daily exposure to the two languages modulates the effect of language as a social categorization cue. This research will allow us to test whether bilingual people use language to categorize individuals belonging to the same sociolinguistic community based on the language these individuals are speaking. Our findings may have relevant political and social implications for linguistic policies in bilingual communities.

## Introduction

Categorization is a fundamental cognitive process worldwide that has the function of organizing and processing stimuli quickly and automatically [1–3]. As human beings, each of us belongs to different social categories: we can be categorized, for instance, as young or old, sporty or non-sporty, parents or non-parents. Social categorization refers to the tendency to classify individuals in terms of the categories they belong or do not belong to. Social categorization is an automatic phenomenon that occurs when we meet a new person and can influence the way we perceive people from different groups [4–6]. Decades of research have been devoted to the study of race, age and gender as the three major cues of social categorization [7–12]. Here we focus on another cue that has received less attention. This is the case of the language used by the interlocutor, which remains unknown until she or he starts speaking. Although people may be able to guess which language is spoken by the interlocutor based on the sociolinguistic contexts they live in, as for instance which language is more frequently used in that context, these guesses can be incorrect. Thus, the language of the interlocutor will only be known (and the guesses confirmed or disconfirmed) when the interlocutor speaks.

Recent studies have shown that infants use language to encode individuals in different groups according to the language they speak. For instance, Kinzler, Dupoux and Spelke [13] observed that 6-month old infants prefer looking at speakers of their same native language than those who speak a different language. Other studies reported that 11- and 19-month old infants, when learning new information, look more frequently at members belonging to the same linguistic group than at people of a different linguistic group [14–16]. These results with the language cue would be analogous to what has been observed with other cues, such as race and gender [17, 18].

Empirical investigations on the role of language as a cue for categorization in adults focused initially on accent, that is, the peculiar pronunciation of a group of individuals from a particular region. Pietraszewski and Schwartz ([19], see also [20]) have exploited the logic underlying the memory confusion paradigm [21, 22], whereby, if an individual’s feature is a cue for categorization, then individuals sharing this feature will be more likely to be confused between each other than between individuals not sharing this feature. In their study, participants were first exposed to pairings of faces and audio statements. Half of the statements were uttered in an English accent (e.g., American accent) and the other half in a different English accent (e.g., British accent). After a brief distractor task, participants were asked to determine which speaker made each statement by selecting the appropriate face from an array containing all the faces. The results showed that when participants incorrectly attributed statements to speakers, they were more likely to choose a speaker with the same accent as the original speaker. That is, participants made more same-accent errors, confusing speakers from the same accent category, than between-accent errors, confusing speakers from the different accent category. These results were interpreted as evidence that accent is a cue for automatic and implicit categorization of faces.

In a recent study Baus, Ruiz-Tada, Escera & Costa [23] have replicated this finding with two different languages instead of two different accents of the same language. Specifically, Spanish participants were exposed to Spanish and English statements. Similar to what was obtained by Pietraszewski and Schwartz [19], same-language errors were more frequent than between-language errors. Interestingly, Baus and colleagues further measured the electrophysiological activity associated to language categorization in an oddball paradigm. The ERP analysis showed an early visual mismatch negativity (vMMN) for between-language category faces, but not for within-category faces. This result seems to indicate that language categorization influences the early stages of face processing. In sum, findings from the memory confusion paradigm suggest that people group individuals (i.e. faces) according to the language (or accent) they speak. Moreover, at the neural level, such categorization is an automatic process able to modulate early visual perceptual processing. The present study aims to define the boundaries of this phenomenon.

One common feature of the studies conducted so far refers to the fact that the accents or languages used in the studies belonged to two different sociolinguistic contexts. For instance, participants in Pietraszewski and Schwartz’s study were American citizens from California who were tested with different English accents, including American, British, or Irish. Thus, the accents tested belonged to two different communities, in this case, two English-speaking countries. Similarly, in the study by Baus and colleagues, participants were Spanish dominant, had English as a foreign language and belonged to a sociolinguistic community where Spanish is an official language while English is not. It is therefore possible that participants are not only categorizing faces according to the accent or language they speak, but they are also categorizing faces mediated by the different sociolinguistic communities to which these faces could be ascribed. Some empirical findings would be congruent with this possibility. It is known that foreign accent generates an immediate classification of the speaker as an out-group member and that such classification activates the stereotypes and stigmas associated to this group [24–28]. Therefore, participants could classify speakers according to the accent or language they speak and/or the sterotypes associated. At the same time, some studies have suggested a role of this kind of social sterotypes on speaker recognition [29–31]. The main aim of the present study will be to explore whether language categorization is a mandatory phenomenon occurring even when the languages associated to the stimuli (i.e., faces) cannot be ascribed to different social communities. To do this we will take advantage of bilingual communities.

People living in a bilingual community are regularly exposed to both single and dual language interaction contexts. More critical for our purposes, an individual from this community may be associated with the two languages used in the community rather with a single language. That is, unlike what normally occurs in monolingual communities where there may be a one-to-one correspondence between interlocutor and language, in bilingual communities there may be a one-to-two correspondence. Interestingly, bilingual speakers seem to be sensitive to this correspondence. Recent studies have shown that bilinguals are able to adapt to language-contexts based on prior knowledge about interlocutors. For instance, Molnar, Ibáñez-Molina and Carreiras [32] familiarized Basque-Spanish bilinguals with three different interlocutors who spoke Spanish, Basque, or both languages. Immediately after the familiarization, participants completed an audio-visual lexical decision task in which the interlocutors produced target words in Spanish or Basque. Reaction times were faster when the language the interlocutors spoke at the lexical decision task matched the language used during familiarization with respect to when the language did not match. In an event-related potential adaptation of Molnar at al.’s study, Martin, Molnar and Carreiras [33] observed that faces associated to one language (i.e., monolingual speakers) elicited a larger early negativity ERP component compared to those associated with two languages (i.e., bilingual speakers). The difference in the ERP deflection was reliable even before the speaker started to speak, suggesting that faces might convey information pertaining to the language(s) associated with the face. These studies suggested that bilinguals are able to anticipate which language their interlocutor will use, congruent with some models of bilingual language control [34].

In the present study, we test whether language automatically functions as a cue for face connotation, even in conditions in which language does not clearly distinguish between different social groups, i.e. when the languages at test belong to the same sociolinguistic context. Participants will be bilingual speakers living in a bilingual community, who are exposed daily to the two languages of their community. We will take advantage of the memory confusion paradigm. If language categorization is an automatic and mandatory process, we expect to replicate previous findings and observe more same-language errors than different-language errors; that is, when participants make an error attributing a statement to a speaker, they are expected to be more likely to choose a speaker of the same language. By contrast, if language categorization is contingent on sociolinguistic categorization, the effect should appear only when languages are ascribed to different social groups, as was the case in the studies by Pietraszewski and Schwartz [19], and Baus et al [23]. Under this latter hypothesis, no language categorization effect should be expected in our studies, where the languages used belong to the same sociolinguistic context in which the bilingual participants are exposed daily to faces speaking those languages.

To obtain a better description of the categorization role of language within bilingual contexts, we will test two different types of bilingual communities. In the first study, we will test Spanish and Basque, two typologically different languages: Spanish is a Romance language from the Indo-European language family while Basque is a non-Indo-European language isolate [35, 36]. Both are co-official languages in the Basque Autonomous Community and Navarre (northeastern Spain). In the second study, we will test two varieties of the Romance language family: Italian and the Veneto dialect [37, 38]. The Veneto dialect is a non-official regional language spoken in Veneto, a north-eastern region of Italy, where the only official language is Italian ([37, 39]; see also [40]). It is possible that in these communities the use of a specific language is associated with different cultural and political sensitivities. For example, the use of Spanish, or Basque, could indicate that the speaker has a different group identification with respect to Spanish and Basque identities; the same situation could happen in relation to the use of Italian or Venetian. If this were the case, instead of, or in addition to language, the participant’s cultural and political sensitivities towards each language could drive the categorization of speakers in our experimental paradimg. To control the impact of this variable, we will use a group identification scale to ensure that our participants are neutral or positive towards Spanish and Basque identities (Study 1) and towards Italian and Venetian identities (Study 2).

A second goal of the current research is to explore whether the language effect on face categorization is modulated by the degree of bilingualism which we operationalize as the amount of participant’s exposure to each of the two languages. In their study, Molnar and colleagues [32] tested two groups of Spanish-Basque bilinguals. One group was composed of balanced (highly proficient) bilinguals who acquired Basque before the age of 3 and reported using both languages on a daily basis with family, friends, and colleagues. The other group was composed of unbalanced (less proficient) Basque-Spanish bilinguals who started learning Basque in school-settings between the age of 9 and 14 and reported using Spanish as the primary language for daily communication. Only balanced bilinguals showed adaptation of their language comprehension processes to the linguistic identity of the interlocutor. Such an effect was not observed in the unbalanced bilinguals’ group. To explore the extent to which language exposure affects the language categorization phenomenon, we will estimate the relative use of each language for each participant and we will add this measure as a continuous predictor to the main analysis (see for a similar procedure, [41]).

In sum, as a main hypothesis, more same-language errors than different-language errors are expected in the two populations of bilinguals. Such a result would be congruent with the assumption that language categorization is an automatic and mandatory process. In addition, in further analyses we will explore whether language exposure modulates this effect.

## Study 1: Spanish-Basque bilinguals

### Materials and methods

#### Participants

Fifty volunteer participants will be recruited. The number of participants satisfied the required sample size based on a statistical power analysis (GPower 3.1; [42]). Statistical power analysis was based on data from Study 1 by Pietraszewski and Schwartz [19]. In that study, 30 participants were tested with the same experimental design as our current study. The correlation index of the paired t-test between Same-accent errors and Different-accent errors was r = 0.78 (t = 6.62; p < .001). With alpha = .05 and power = 0.95, the anticipated sample size required to obtain a significant effect is n = 25. In contrast to the study by Pietraszewski and Schwartz [19], our study will be an online study. Recent studies have validated psychological research based on internet samples [43, 44]. However, following Brysbaert’s suggestion [45], we have decided to run a more well-powered study than the original one and we will double the sample size required. Moreover, we would like to evaluate the impact of degree of bilingualism as a continuous variable. This type of analysis needs larger samples. All participants will be required to give written informed consent, and all experimental procedures were approved by the local Research Ethics Committees of the University of Padova (Protocol number: 3589; Title: *The social bilingual brain*). All data will be made available under the following OSF repository: https://osf.io/3fudg/.

At the end of the main experimental session, participants will be required to provide personal information using a questionnaire which consists of four parts: a) *general information* concerning the language the participant used as a child and the age of acquisition; b) *perceived proficiency*, in which the participant rates their degree of perceived proficiency in comprehension and production using a 1–10 point scale (1 = “one”; 10 = “perfect”) in both languages; c) *language use*, in which the participant quantifies the use of each language in various daily activities; and d) *group identification*, where the participant’s level of identification with their groups (i.e., Spanish/Basque or Italian/Venetian) is assessed in 4 questions using a 1–7 point scale (1 = “not at all”; 7 = “very much”).

In order to ensure that our participants are highly proficient and able to interact in both languages, only results of those participants with a mean >6 in part b of the questionnaire (*perceived proficiency)* in both languages will be analyzed. A Relative Use Index will be calculated for each participant applying the following formula to the daily activities answered in part c of the questionnaire (*language use)*: (value in language A—value in language B) / (value in language A + value in language B). The mean between the scores obtained in all daily activities will correspond to the Relative Use Index for a particular individual. This ratio will score from -1 to 1. The value of 0 indicates a perfectly balanced bilingual, that is, with a similar amount of use of the two languages. Positive or negative values indicate the inclination of use towards one language or the other. Finally, to control for participants’ level of identification with the groups, four questions are added in part d of the questionnaire (*group identification*). These questions are based on research by Latrofa, Vaes, Pastore & Cadinu [46] and aim to evaluate the group level of identification of our participants (Spanish and Basque, and Italian and Venetian for Studies 1 and 2 respectively). In order to make sure that our participants are neutral or positive towards both groups of their communities, we reject from the analysis those participants with a mean score < 3 in both *group identification* scales. The full questionnaire can be consulted on the platform OSF (https://osf.io/3fudg/).

#### Materials

Eight gray-scale photographs of male Caucasian faces were taken from Martinez & Benavente [47]. All of them were emotionally neutral and had no extra visual details. Twenty-four non-autobiographical sentences were created and then recorded in Spanish and Basque using the software Audacity (v 2.0.3) (e.g., *La tienda se queda vacía*—*Dendahutsikgeratu da*; “The store becomes empty”, in Spanish and Basque, respectively). The differences in length between Spanish and Basque sentences were measured by calculating the number of sounds because words are not a good unit for comparing Spanish and Basque. Indeed, Basque is an agglutinative language, and all determiners and prepositions are embedded with their nouns, while in Spanish determiners and prepositions are written separately. The number of sounds did not diverge between Spanish [mean = 19.58 sounds, range = 13–25] and Basque [mean = 20 sounds, range = 12–22] (t < 1) sentences. Recording durations for sentences in Spanish [mean = 1.91 seconds, range = 1.52–2.48] and Basque [mean = 1.84 seconds, range = 1.05–2.49] did not differ (t(46) = 0.79, p = 0.42). Four male native Spanish speakers and four male native Basque speakers recorded the sentences. The final design consisted of photographs of faces accompanied by a voice speaking either in Spanish or in Basque. Sixteen lists were created to counterbalance the face, sentence and language. Therefore, all faces accompanied every sentence in both languages across all participants. The sentences together with the considered control variables can be consulted on the platform OSF (https://osf.io/3fudg/).

#### Procedure

**Fig 1:**
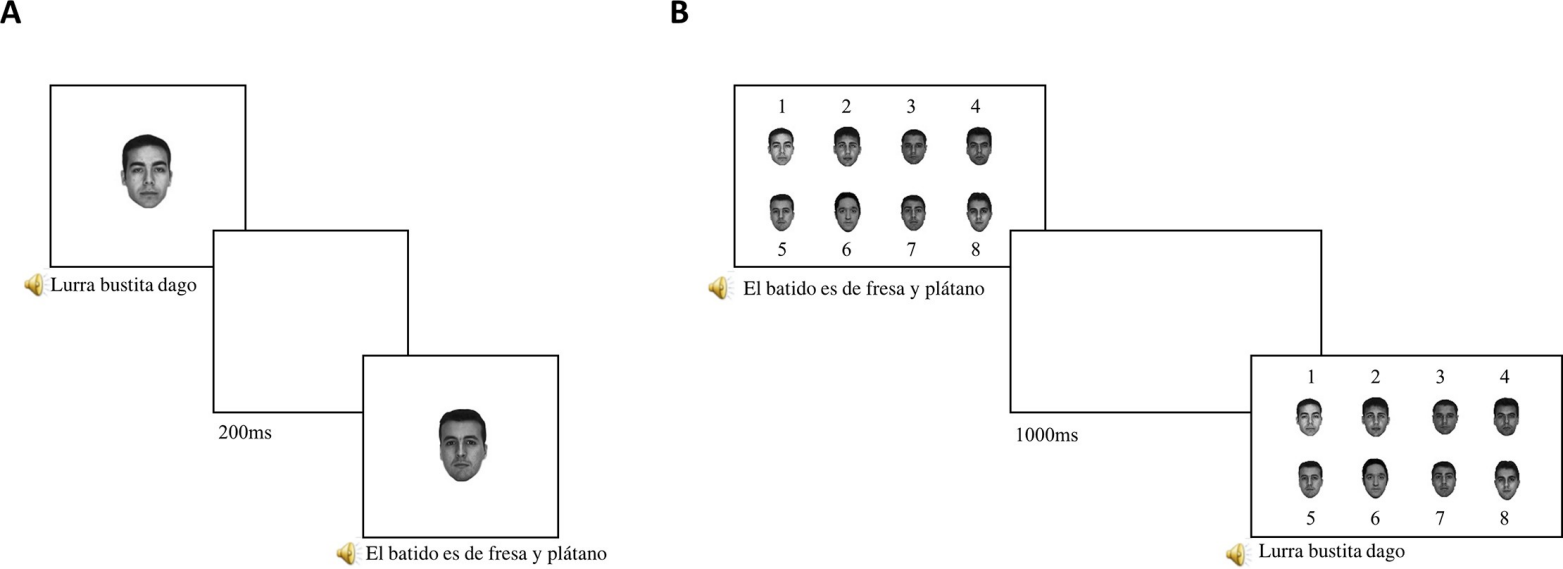
The procedure of the Memory Confusion Paradigm (MCP). This diagram shows the two phases of the MCP. On Panel A, the encoding phase, where faces were presented with the audio sentences. On panel B, the final recognition phase. Grayscale photos of eight Caucasian males with neutral expressions were selected from the free AR face database [47].

The experiment consists of four sessions: the encoding phase, the distractor task (*tetris game*), the recognition phase and finally, the questionnaire described above. At the beginning of the experiment, the participant is only aware of the first session and will be informed that the study will take approximately 15 minutes. In the first phase, the encoding phase, face photographs will be presented on the screen one at a time along with the auditory presentation of the sentences. Participants will only be asked to form impressions about the speakers as they watch and listen because they will then be asked questions about them. Trial structure will be the following: one photo and audio are presented simultaneously on the screen. Each speaker’s photo will be displayed for the entire duration of the statement, plus two additional seconds thereafter, followed by a blank presented on the screen for 200ms (Fig 1). Each of the 8 faces will be presented 3 times during the coding phase, for a total of 24 presentations. The three presentations of each face will have three different sentences, but the voice will be the same. In other words, each face will be paired with the same voice and will be associated with three different sentences. The language of the sentences in the first two positions will be counterbalanced between the lists so that 8 lists will start with two Spanish sentences and 8 with two Basque sentences. Language order will be unsystematic thereafter, within the constraint that each speaker spoke once during statements 1–8, once again in statements 9–16, and once in statements 16–24. Upon completion of the encoding phase, participants will be engaged in a distractor filler task (Tetris game) for 2 minutes to avoid having the recognition phase immediately after the encoding phase.

After that, participants will start the second phase of the Memory Confusion paradigm, the recognition phase, in which all 8 photographs are presented on the screen, numbered from 1 to 8. Face order will be randomized across trials. Then, the same 24 sentences of the encoding phase will be presented again in auditory form. The participant must decide which of the 8 faces accompanied the sentence in the encoding phase by clicking on the corresponding number. The eight faces will remain on the screen until the participant’s response, after which a blank lasting 1000 ms will be presented (see Fig 1). This procedure continues until all 24 sentences of the encoding phase have been presented. The experiment will last about fifteen minutes.

After the recognition phase, participants will complete the questionnaire. At the end of the experimental session participants will be thanked and debriefed by describing the real aims of the experiment. In addition, participants will again be asked for their consent for their results to be used.

#### Methodology for data collection

The experiment will take place online, through the *Ibexfarm* platform. Participants will be able to access the test by clicking on a link. Participants will be recruited through the participant pool database of The Bilingual Mind research group for Study 1, while for Study 2 a ‘snowball’ procedure will be used through social media.

#### Methodology for analysis

First, to test for the presence of a Language effect, categorization will be measured on a participant basis by calculating the difference in error rates between same-language errors and different-language errors. While there are only three possibilities to make same-language errors (because one of the faces is the correct answer), there are four possibilities to make a different-language error. To correct for this discrepancy, the number of between language errors will be multiplied by 0.75. Following previous studies that have used this paradigm [19, 20], paired t-test analyses will be performed between same-language and different-language errors (see [48] for validation of this method). In addition, to explore the influence of language exposure on the language categorization effect, the Relative Use Index will be added as a covariate in the paired t-tests.

Moreover, being an online experiment, it is important to control for participant’s performance during the task. To this end, reaction time measures in the recognition phase will be collected as a control measure. More specifically, the time participants require to decide which face corresponds to the sentence will be recorded. These response times will serve to assess the participant’s level of engagement in the task. Participants with a mean response time faster or slower than 2.5 standard deviation of the mean group will be considered outliers and removed from the analysis. Additionally, although previous studies did not measure response time, we will be able to explore whether participants are slower selecting incorrect than correct faces as well as whether response time differences are revealed for incorrect ingroup face selection as compared to incorrect outgroup face selection.

As a sanity check to control whether the memory confusion paradigm is doing what it is supposed to do, we expect error rates to be high. In particular, according to the previous literature [19, 20] error rates should be greater than 50%.

#### Predictions

Assuming that linguistic categorization is an automatic and mandatory process, we predict more same-language errors than different-language errors in the two populations of bilinguals. That is, when participants make an error attributing a statement to a speaker, they are expected to be more likely to choose a speaker of the same language. In addition, based on a previous study [32], we predict a positive correlation between the Relative Use Index covariate and categorization.

## Study 2: Italian-Venetian bilinguals

### Materials and methods

#### Participants

Fifty Italian-Venetian participants will be recruited.

#### Materials

The same eight gray-scale photographs of male Caucasian faces as in Study 1 will be used in Study 2. Twenty-four non-autobiographical sentences were created and then recorded in Italian and Venetian (*Il pane fresco è finito*—*El pan fresco l’è finio;* “The fresh bread is finished”, in Italian and Venetian, respectively) using the software Audacity (v 2.0.3). Sentences’ word length did not diverge between Italian [mean = 5.45 words, range = 4–8] and Venetian [mean = 5.58 words, range = 4–8] (t < 1). Four male native Italian speakers and four male native Venetian speakers recorded the sentences. Recording durations for sentences in Italian [mean = 2.01 seconds, range = 1.44–2.52] and Venetian [mean = 1.91 seconds, range = 1.35–2.79] did not differ (t(46) = 1.01, p = 0.31). The final design and list were identical to Study 1. The sentences along with the control variables considered can be consulted through the platform OSF (https://osf.io/3fudg/).

#### Procedure

Identical to Study1.

#### Methodology for data collection

Identical to Study1.

#### Methodology for analysis

Identical to Study1.

#### Predictions

Identical to Study1.
